# The Impact of tagSNPs in CXCL16 Gene on the Risk of Myocardial Infarction in a Chinese Han Population

**DOI:** 10.1155/2017/9463272

**Published:** 2017-02-14

**Authors:** Shun Xu, Jie Cheng, Meng-yun Cai, Li-li Liang, Jin-ming Cen, Xi-li Yang, Can Chen, Xinguang Liu, Xing-dong Xiong

**Affiliations:** ^1^Institute of Aging Research, Guangdong Medical University, Dongguan, China; ^2^Guangdong Provincial Key Laboratory of Medical Molecular Diagnostics, Guangdong Medical University, Dongguan, China; ^3^Institute of Biochemistry & Molecular Biology, Guangdong Medical University, Zhanjiang, China; ^4^Department of Clinical Laboratory, The Affiliated Hospital of Guangdong Medical University, Zhanjiang, China; ^5^Department of Cardiovascular Disease, The First People's Hospital of Foshan, Foshan, China; ^6^Department of Cardiovascular Disease, The Affiliated Hospital of Guangdong Medical University, Zhanjiang, China

## Abstract

*CXCL16 *has been demonstrated to be involved in the development of atherosclerosis and myocardial infarction (MI). Nonetheless, the role of the* CXCL16* polymorphisms on MI pathogenesis is far to be elucidated. We herein genotyped four tagSNPs in* CXCL16* gene (rs2304973, rs1050998, rs3744700, and rs8123) in 275 MI patients and 670 control subjects, aimed at probing into the impact of* CXCL16 *polymorphisms on individual susceptibility to MI. Multivariate logistic regression analysis showed that C allele (OR = 1.31, 95% CI = 1.03–1.66, and *P* = 0.029) and CC genotype (OR = 1.84, 95% CI = 1.11–3.06, and *P* = 0.018) of rs1050998 were associated with increased MI risk; and C allele (OR = 0.77, 95% CI = 0.60–0.98, and *P* = 0.036) of rs8123 exhibited decreased MI risk, while the other two tagSNPs had no significant effect. Consistently, the haplotype rs2304973T-rs1050998C-rs3744700G-rs8123A containing the C allele of rs1050998 and A allele of rs8123 exhibited elevated MI risk (OR = 1.41, 95% CI = 1.02–1.96, and *P* = 0.037). Further stratified analysis unveiled a more apparent association with MI risk among younger subjects (≤60 years old). Taken together, our results provided the first evidence that* CXCL16 *polymorphisms significantly impacted MI risk in Chinese subjects.

## 1. Introduction

Myocardial infarction (MI), a main manifestation of coronary artery disease (CAD), poses increasing pressure on public health worldwide. Numerous environmental factors, such as obesity, hypercholesterolemia, alcohol intake, smoking, diabetes, and hypertension, have been established to contribute to the development of MI [1, 2]. Moreover, in addition to these modifiable factors, there is a growing body of studies having focused on the influence of genetic variants or polymorphisms within candidate genes in MI pathogenesis and thus yielding accumulating evidences that polymorphic variants in host genes exert crucial roles on the risk of MI [3, 4].

*CXCL16*, a newly discovered cytokine belonging to the CXC chemokine family, is expressed in both transmembrane and soluble forms [5]. As a transmembrane molecule,* CXCL16* (also known as* SR-PSOX*) acts as a scavenger receptor for oxidized low-density lipoprotein (oxLDL) uptake, suggesting the involvement of* CXCL16* in lipid metabolism [6]. While in a soluble form,* CXCL16 *has been found to interact with its receptor, CXCR6, and thus functions as an attractant and adhesion molecule for CXCR6-expressing T cells, which contribute to the development of atherosclerosis [7, 8]. Mounting evidences have uncovered the close association of* CXCL16* with the development of diverse human inflammatory diseases, including atherosclerosis [9], coronary artery disease [10], and MI [11]. Enhanced expression of both* CXCL16 *and* CXCR6* has been observed in atherosclerotic lesions from humans as well as from apolipoprotein E- (apoE-) deficient mice [12]. And the elevated expression level of* CXCL16* was observed in MI patients as well [11]. Moreover, it has been reported that soluble CXCL16 in plasma could serve as a biomarker for acute coronary syndromes [13]. Thus it was reasonable to speculate that* CXCL16 *polymorphisms might probably exert an important role in MI pathogenesis.

Though the association between* CXCL16 *and MI pathogenesis has been fully studied, the effect of polymorphic variants in* CXCL16* gene on the individual susceptibility to MI and its underlying molecular mechanisms are far to be elucidated. Thus, we herein conducted a case-control study to explore the association of the four tagSNPs in* CXCL16* genes (rs2304973, rs1050998, rs3744700, and rs8123) with the risk of MI. Our data unraveled that the C allele of rs1055998 and the haplotype rs2304973T-rs1050998C-rs3744700G-rs8123A conferred an increased risk of MI in the Chinese Han population.

## 2. Materials and Methods

### 2.1. Study Subjects

A total of 945 Chinese Han subjects (275 MI patients and 670 control subjects) were included in our case-control study, who were consecutively recruited from the Affiliated Hospital of Guangdong Medical University (Zhanjiang, China) and the First People's Hospital of Foshan (Foshan, China) from March 2011 to December 2015. The diagnosis of MI was described previously [3]. 670 control subjects were recruited for regular physical examinations during the same period when MI patients were recruited. The 670 control subjects were judged to be free of MI by questionnaires, medical history, clinical examination, and electrocardiography. The individuals with a history of hematologic, renal, neoplastic, liver, or thyroid diseases were excluded.

All study subjects were genetically unrelated ethnic Han Chinese. Each subject was interviewed to collect information on demographic data and risk factors related to MI after obtaining the informed consent. The study was approved by the Medical Ethics Committee of the First People's Hospital of Foshan and the Affiliated Hospital of Guangdong Medical University.

### 2.2. Biochemical Parameters Analysis

An approximately 2 mL peripheral blood sample was drawn from each subject into tubes containing ethylenediaminetetraacetic acid (EDTA) after obtaining the informed consent. Immediately after collection, the blood sample was centrifuged at 2000 ×g for 15 min and stored at −80°C. The levels of plasma total cholesterol (TC), triglyceride (TG), high density lipoprotein cholesterol (HDL-C), and low-density lipoprotein cholesterol (LDL-C) were measured enzymatically using a chemistry analyzer (Olympus, Japan). Glucose was analyzed by the glucose oxidase method with an Abbott V/P Analyzer (Abbott Laboratories, USA).

### 2.3. DNA Extraction

Genomic DNA was extracted from peripheral whole blood utilizing TIANamp blood DNA extraction kit (TianGen Biotech, China) according to the manufacturer's recommendations. All DNA samples were dissolved in water and stored at −20°C until use.

### 2.4. TagSNP Selection and Genotyping

**Figure 1:**
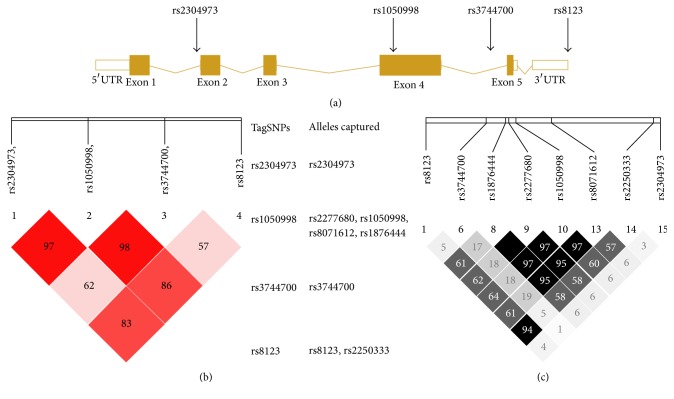
Schematic of* CXCL16* gene structure and pairwise LD between the four tagSNPs. (a) Schematic of the* CXCL16* gene structure and the location of the four tagSNPs (rs2304973, rs1050998, rs3744700, and rs8123) within CXCL16 gene. (b) *D*′ values are plotted as a graph to show linkage disequilibrium among the four tagSNPs. Details of the selected tagSNPs and respective SNPs captured by the four tagSNPs are also indicated. (c) The *r*^2^ values are plotted as a graph to show the linkage disequilibrium measure among the eight SNPs (rs8123, rs3744700, rs1876444, rs2277680, rs1050998, rs8071612, rs2250333, and rs2304973) captured by these four tagSNPs within CXCL16 gene.

The Chinese Han population's SNP data of* CXCL16* gene were downloaded from the HapMap database (http://www.hapmap.org). Then the SNP data of* CXCL16* gene were analyzed using Haploview software version 4.2 [14] and obtained four tagSNPs, including rs2304973, rs1050998, rs3744700, and rs8123 (Figure 1(a)). A minor allele frequency (MAF) > 0.05 and a linkage disequilibrium measure (*r*^2^) > 0.8 were prerequisites for tagSNPs selection (*r*^2^ values were shown in Figure 1(c)). These four tagSNPs would capture the information of the 8 known* CXCL16* SNPs with a MAF > 0.05 (Figure 1(b)). Furthermore, the haplotypic blocks of the four tagSNPs were performed with the SHEsis platform [15].

The genotyping of the four tagSNPs was performed utilizing polymerase chain reaction-ligase detection reaction (PCR-LDR) method (Shanghai Biowing Applied Biotechnology Company), as described previously [16]. The sequences of primers and probes were listed in Table S1 in Supplementary Material available online at https://doi.org/10.1155/2017/9463272.

### 2.5. Statistical Analysis

All the four tagSNPs of* CXCL16* gene were tested for confirmation using Hardy-Weinberg expectations by a goodness-of-fit *χ*^2^ test among the control subjects. Quantitative variables were expressed as mean ± standard deviation (SD), and qualitative variables were expressed as percentages. The differences of the demographic characteristics between the cases and controls were estimated by the *χ*^2^ test (for categorical variables) and Student's* t-*test (for continuous variables).

For the association analysis of individual tagSNP with MI risk, genotype frequencies were assessed by means of multivariate methods based on logistic regression analysis. And the odds ratios (ORs) and 95% confidence intervals (CIs) for the effect of SNPs on MI risk were adjusted by age, sex, smoking, drinking, hypertension, diabetes, and hyperlipidemia. The statistical analyses were performed using the SPSS software (version 19). The haplotype analysis on the polymorphisms was done using SHEsis software freely available at (http://analysis.bio-x.cn/myAnalysis.php). *P* value of less than 0.05 was used as the criterion of statistical significance.

## 3. Results

### 3.1. Characteristics of the Study Population

The characteristics of the enrolled subjects in the study (275 MI cases and 670 control subjects) were listed in Table 1. In comparison with control subjects, the MI patients exhibited higher proportion of male gender, smokers, and alcohol consumers (*P* < 0.001, *P* < 0.001, and *P* < 0.001, resp.), more prevalence of hypertension, hyperlipidemia and diabetes (*P* < 0.001, *P* < 0.001, and *P* < 0.001, resp.), and higher levels of fasting plasma glucose (FPG), triglycerides (TG), and LDL-C (*P* < 0.001, *P* < 0.001, and *P* < 0.001, resp.) but lower HDL-C (*P* < 0.001), while no statistically significant difference between cases and controls was observed in terms of age (*P* = 0.483) and TC levels (*P* = 0.175). In all, these data further demonstrated that male gender, smoking, alcohol intake, hypertension, hyperlipidemia, and diabetes mellitus were the critical risk factors for MI development in Chinese population.

### 3.2. Multivariate Associations of Four tagSNPs with the Risk of MI

Four tagSNPs (rs2304973, rs1050998, rs3744700, and rs8123) in* CXCL16* gene were genotyped in 275 MI patients and 670 control subjects. The primary information for these four polymorphisms was listed in Table 2. Minor allele frequency (MAF) of all four tagSNPs in the control subjects was similar to MAF for Chinese in HapMap database (Table 2). All the genotype frequency distributions of the four tagSNPs in the controls followed Hardy-Weinberg equilibrium proportions (all* P *values ≥ 0.10, Table 2).

The allele and genotype distributions of the four tagSNPs in the cases and controls were presented in Table 3. The allelic association analysis uncovered that the C allele of rs1050998 was associated with an evidently enhanced risk of MI (OR = 1.31, 95% CI = 1.03–1.66, and *P* = 0.029, Table 3). In addition, compared to TT genotype, the CC homozygote (OR = 1.84, 95% CI = 1.11–3.06, and *P* = 0.018, Table 3) or CT heterozygote (OR = 1.67, 95% CI = 1.03–2.70, and *P* = 0.037, Table 3) exhibited an increased risk of MI as well. Moreover, the C allele of rs8123 conferred a diminished risk of MI compared to A allele (OR = 0.77, 95% CI = 0.60–0.98, and *P* = 0.036, Table 3). Consistently, the CC as well as combined AC + CC genotypes showed borderline significantly decreased risk for MI (Table 3, OR = 0.58, 95% CI = 0.34–1.01, and *P* = 0.054 and OR = 0.73, 95% CI = 0.52–1.02, and *P* = 0.065, resp.), in comparison with the GG genotype. In all, our data indicated that* CXCL16* tagSNPs were closely associated with MI risk in the Chinese Han population. And individuals carrying C allele of rs1050998 exhibited significantly increased MI susceptibility, while the C allele of rs8123 potentially provided a protective effect against MI risk. However, no significant association between rs2304973 and rs3744700 and MI risk was observed (Table 3).

### 3.3. Stratification Analyses of* CXCL16* rs1050998 and rs8123 Polymorphism and Risk of MI

We further evaluated the alleles or genotypes of* CXCL16 *rs1050998 and rs8123 and MI susceptibility after stratifying the subjects by age, sex, status of smoking, or drinking. Stratification analyses by age (≤60 or >60 years old) unveiled that the increased MI risk of individuals carrying C allele (OR = 1.59, 95% CI = 1.08–2.35, and *P* = 0.020, Table 4) or CC genotype (OR = 2.49, 95% CI = 1.11–5.59, and *P* = 0.027, Table 4) of rs1050998 was more notable among younger subjects (≤60 years old) whereas no significant association was observed from the group older than 60 years old (Table 4). And the protective effect of C allele (OR = 0.60, 95% CI = 0.40–0.89, and *P* = 0.012, Table 4) or CC genotype (OR = 0.38, 95% CI = 0.16–0.93, and *P* = 0.031, Table 4) of rs8123 was more evident among younger subjects (≤60 years old) as well. No more significant association between* CXCL16 *rs1050998 and rs8123 polymorphism and risk of MI was observed among subgroups by sex, status of smoking, or drinking (data not shown).

### 3.4. Association between the Haplotypes of* CXCL16* tagSNPs with the Risk of MI

As shown in Figure 1(b), all the four tagSNPs were located in one haplotypic block. We thus further compared the haplotype frequencies of the four tagSNPs between MI group and controls. Four common haplotypes (frequency > 3%) derived from the four tagSNPs accounted for approximately 96% of the haplotype variations (Table 5). Consistently, among the four common haplotypes, the haplotype containing C allele of rs1050998 and A allele of rs8123 (rs2304973T-rs1050998C-rs3744700G-rs8123A) was found to be associated with an increased risk for MI (OR = 1.41, 95% CI = 1.02–1.96, and *P* = 0.037, Table 5); and the haplotype containing T allele of rs1050998 and C allele of rs8123 (rs2304973C-rs1050998T- rs3744700G-rs8123C) exhibited a reduced MI risk (OR = 0.77, 95% CI = 0.62–0.96, and *P* = 0.022, Table 5).

## 4. Discussion

Previous studies have established the close association between* CXCL16* and the pathogenesis of atherosclerosis and MI. Nonetheless, the impact of tagSNPs in* CXCL16* gene on MI risk is still largely unknown. In this study, we performed a genetic association analysis on the four tagSNPs (rs2304973, rs1050998, rs3744700, and rs8123) within* CXCL16* gene and unraveled that the C allele of rs1050998 and the A allele of rs8123 and the haplotype rs2304973T-rs1050998C-rs3744700G-rs8123A containing C allele of rs1050998 and the A allele of rs8123 conferred enhanced risk of MI in the Chinese Han population. Moreover, the association between* CXCL16* polymorphisms and MI risk was more remarkable among younger subjects (≤60 years old). These data indicated that the C allele of rs1050998 and the A allele of rs8123 might significantly enhance the risk of MI in the Chinese Han population.

The association of polymorphisms of the* CXCL16* gene locus with various inflammatory diseases has been widely studied [17]. However, the effect of* CXCL16 *tagSNPs on MI risk is still unknown. Zivković et al. have reported that the rs1050998 (I142T) polymorphisms were significantly associated with the occurrence of Carotid Atherosclerosis (CA) plaque (OR = 1.27, *P* = 0.03) [9]. Our data indicated that individuals carrying C allele of rs1050998 exhibited enhanced MI risk, which was consistent with the previously published literature [9]. Another study suggested that the rs2304973 showed no significant difference between CAD patients and control subjects [18], which was compatible with our results that there is no evident association of rs2304973 with the risk of MI. In addition, the rs3744700 polymorphism has been reported to be closely related to the development of CAD (OR = 1.77, *P* < 0.001) [18, 19]; however, there is no significant association between rs3744700 and MI risk in our case-control study, which might be due to the difference between CAD and MI.

As shown that both rs1050998 and rs8123 tagSNPs capture other closely linked SNPs (high LD) within or near the* CXCL16* gene locus (Figure 1), thus the association of rs1050998 and rs8123 polymorphisms with MI risk might be direct due to their causative effect or because of the other functional polymorphisms captured by them. The rs2277680 polymorphism captured by rs1050998 has been unveiled to have a marginal association with the risk of Crohn's disease (CD) in patients (*P* = 0.0482, OR = 1.4310) [20] but exhibited no significant association with CAD risk [18]. Similarly, no significant difference was observed for the distribution of the rs2250333 polymorphism captured by rs8123 between CAD patients and control subjects as well [18]. We noticed that the* CXCL16* rs1050998 (T/C) polymorphism caused the T-to-C change, which resulted in the missense mutation of I (Ile) 142T (Thr). One single amino acid mutation might extensively impact the structure, stability, and activity of the protein [21–23], especially when the amino acid changed between nonpolar amino acid (Ile) and polar amino acid (Thr) [24]. Thus, it is reasonable to speculate that the rs1050998 polymorphism might exert a direct causative effect on the MI risk.

The stratified analyses of the association of rs1050998 and rs8123 polymorphisms with MI risk revealed that the increased risk of* CXCL16* rs150998 and rs8123 in MI was more remarkable among younger subjects (≤60 years old), while no significant association was observed in the older group (>60 years old) (Table 4). This phenomenon was similar to our previous study, which uncovered that the enhanced risk conferred by* LRP6* rs rs2302685 in MI was more evident among younger subjects (≤60 years old) as well [3]. The potential explanation to this phenomenon was that the dominant cause of MI pathogenesis in older subjects is more likely due to the aging effects rather than direct genetic effects.

There are several limitations in this case-control study that need to be addressed. Initially, the possibility that the subjects (275 MI patients and 670 control subjects) enrolled from hospitals may not represent the general population could not be excluded. Nevertheless, the distributions of the selected tagSNPs in the controls were in Hardy-Weinberg equilibrium. Second, the relatively small sample size limited the statistical power of this study, especially for the case subjects. Finally, further investigations in different population and with larger sample size contribute to verifying the general validity of our findings. However, the results drawn from our case-control study provided novel insights and fascinating information for further studies in this area.

## 5. Conclusions

Taken together, our case-control study firstly provides the evidences that the* CXCL16* polymorphisms significantly impacted the risk of MI in the Chinese Han population, and the association between* CXCL16* polymorphisms and MI risk was more evident among younger subjects.

## Supplementary Material

Table S1 showed the sequences of all the primers and probes used to genotype the four tagSNPs.

## Figures and Tables

**Table 1:** The characteristics of MI cases and controls.

Variable	Controls (*n* = 670)	Cases (*n* = 275)	*P* ^a^
Age (years)	61.48 ± 12.31	62.10 ± 12.00	0.483
Sex (male)	387 (57.8%)	213 (77.5%)	**<0.001** ^b^
Smoking	174 (26.0%)	163 (59.3%)	**<0.001**
Drinking	95 (14.2%)	73 (26.5%)	**<0.001**
Hypertension	239 (35.7%)	171 (62.2%)	**<0.001**
Diabetes	107 (16.0%)	129 (46.9%)	**<0.001**
Hyperlipidemia	253 (37.8%)	195 (70.9%)	**<0.001**
Systolic BP (mmHg)	132.56 ± 18.91	140.53 ± 18.77	**<0.001**
Diastolic BP (mmHg)	72.97 ± 10.47	75.93 ± 10.87	**<0.001**
FPG (mM)	5.80 ± 1.88	6.61 ± 1.70	**<0.001**
Triglycerides (mM)	1.49 ± 0.81	2.07 ± 0.96	**<0.001**
Total cholesterol (mM)	4.62 ± 1.15	4.73 ± 1.19	0.175
LDL cholesterol (mM)	2.64 ± 0.90	3.04 ± 0.97	**<0.001**
HDL cholesterol (mM)	1.37 ± 0.66	1.20 ± 0.40	**<0.001**

^a^Two-sided chi-square test or independent-samples *t*-test.

^b^ *P* values under 0.05 were indicated in bold font.

**Table 2:** Primary information for rs2304973, rs1050998, rs3744700, and rs8123 SNPs.

Genotyped SNPs	rs2304973	rs1050998,	rs3744700	rs8123
Chr Pos (Genome Build 107.0)	4738927	4735442	4734715	4733270
Pos in Cxcl16 gene	Intron 1	Extron 4	Intron 4	nearGene-3
MAF^a^ for Chinese (CHB) in HapMap	0.089	0.451	0.134	0.291
MAF in our controls (*n* = 670)	0.089	0.437	0.110	0.369
*P* value for HWE^b^ test in our controls	0.540	0.281	0.444	0.370

^a^MAF: minor allele frequency.

^b^HWE: Hardy-Weinberg equilibrium.

**Table 3:** Multivariate associations of the four tagSNPs in *CXCL16* gene with the risk of MI.

Type	Controls (*n* = 670)	Cases (*n* = 275)	OR (95% CI)^a^	*P* value^a^
	Number (%)	Number (%)		
*rs2304973*				
C	1221 (91.1)	485 (88.2)	1.00	—
T	119 (8.9)	65 (11.8)	1.46 (1.00–2.12)	0.050
CC	555 (82.8)	216 (78.5)	1.00	—
CT	111 (16.6)	53 (19.3)	1.31 (0.85–2.00)	0.220
TT	4 (0.6)	6 (2.2)	4.16 (0.96–18.03)	0.057
CC	555 (82.8)	216 (78.5)	1.00	—
CT + TT	115 (17.2)	59 (21.5)	1.44 (0.96–2.17)	0.080
*rs1050998*				
T	586 (43.7)	215 (39.1)	1.00	—
C	754 (56.3)	335 (60.9)	1.31 (1.03–1.66)	**0.029**
TT	135 (20.1)	38 (13.8)	1.00	—
CT	316 (47.2)	139 (50.5)	1.67 (1.03–2.70)	**0.037**
CC	219 (32.7)	98 (35.6)	1.84 (1.11–3.06)	**0.018** ^b^
TT	135 (20.1)	38 (13.8)	1.00	—
CT + CC	535 (79.9)	237 (86.2)	1.74 (1.10–2.75)	**0.018**
*rs3744700*				
G	1193 (89.0)	487 (88.5)	1.0	—
T	147 (11.0)	63 (11.5)	1.05 (0.72–1.51)	0.811
GG	533 (79.6)	216 (78.5)	1.0	—
GT	127 (19.0)	55 (19.3)	1.02 (0.68–1.56)	0.905
TT	10 (1.5)	4 (1.5)	0.80 (0.19–3.46)	0.767
GG	533 (79.6)	216 (78.5)	1.0	—
GT + TT	137 (20.4)	59 (21.5)	1.04 (0.69–1.56)	0.857
*rs8123*				
A	845 (63.1)	377 (68.5)	1.00	—
C	495 (36.9)	173 (31.5)	0.77 (0.60–0.98)	**0.036**
AA	269 (40.1)	130 (47.3)	1.00	—
AC	307 (45.8)	117 (42.5)	0.78 (0.55–1.11)	0.163
CC	94 (14.0)	28 (10.2)	0.58 (0.34–1.01)	0.054
AA	269 (40.1)	130 (47.3)	1.00	—
AC + CC	401 (59.9)	145 (52.7)	0.73 (0.52–1.02)	0.065

^a^Adjusted for age, sex, smoking, drinking, hypertension, diabetes, and hyperlipidemia.

^b^ *P* values under 0.05 were indicated in bold font.

**Table 4:** Multivariate associations of the rs1050998 and rs8123 in *CXCL16* gene with the risk of MI by further stratification for age.

Genotype	Age ≤ 60		Age > 60	
	OR (95% CI)^a^	*P* value^a^	OR (95% CI)^b^	*P* value
*rs1050998*				
T	1.00	—	1.00	—
C	1.59 (1.08–2.35)	**0.020** ^b^	1.19 (0.87–1.61)	0.273
TT	1.00	—	1.00	—
CT	1.50 (0.70–3.24)	0.300	1.78 (0.94–3.36)	0.075
CC	2.49 (1.11–5.59)	**0.027**	1.60 (0.83–3.08)	0.161
*rs8123*				
A	1.00	—	1.00	—
C	0.60 (0.40–0.89)	**0.012**	0.87 (0.63–1.19)	0.377
AA	1.00	—	1.00	—
AC	0.56 (0.32–0.98)	**0.041**	0.91 (0.57–1.46)	0.701
CC	0.38 (0.16–0.93)	**0.031**	0.72 (0.36–1.45)	0.359

^a^Adjusted for sex, smoking, drinking, hypertension, diabetes, and hyperlipidemia.

^b^ *P* values under 0.05 were indicated in bold font.

**Table 5:** Association between haplotypes of the four tagSNPs in* CXCL16 *gene with the risk of MI.

Haplotype^a^	Controls (*n* = 670)	Cases (*n* = 275)	OR (95% CI)	*P*
	Number (%)	Number (%)		
C C G A	604.21 (45.1)	257.74 (46.9)	1.08 (0.88–1.32)	0.482
C T G C	438.97 (32.8)	150.95 (27.4)	0.77 (0.62–0.96)	**0.022** ^b^
C T T A	126.76 (9.5)	54.96 (10.0)	1.06 (0.76–1.48)	0.772
T C G A	112.85 (8.3)	63.25 (11.5)	1.41 (1.02–1.96)	**0.037**

^a^The allelic sequence in the haplotypes is in the following order: rs2304973, rs1050998, rs3744700, and rs8123.

^b^ *P *values under 0.05 were indicated in bold font.

## Abbreviations

CXCL16: CXC motif chemokine ligand 16
SNP: Single nucleotide polymorphism
MI: Myocardial infarction
CAD: Coronary artery disease
PCR-LDR: Polymerase chain reaction-ligase detection reaction
OR: Odds ratio
CI: Confidence interval
LDL-C: Low-density lipoprotein cholesterol
TC: Total cholesterol
TG: Triglyceride
HDL-C: High density lipoprotein cholesterol.

## References

[1] Zhang X.-H., Lu Z. L., Liu L. (2008). Coronary heart disease in China. *Heart*.

[2] Ramaraj R. (2009). Risk factors for myocardial infarction in women and men. *European Heart Journal*.

[3] Xu S., Cheng J., Chen Y.-N. (2014). The LRP6 rs2302685 polymorphism is associated with increased risk of myocardial infarction. *Lipids in Health and Disease*.

[4] Juan Z., Wei-Guo Z., Heng-Liang S., Da-Guo W. (2015). Association of matrix metalloproteinase 9 C-1562T polymorphism with genetic susceptibility to myocardial infarction: a meta-analysis. *Current Therapeutic Research - Clinical and Experimental*.

[5] Norlander A. E., Saleh M. A., Madhur M. S. (2013). CXCL16: a chemokine-causing chronic kidney disease. *Hypertension*.

[6] Jansson A. M., Aukrust P., Ueland T. (2009). Soluble CXCL16 predicts long-term mortality in acute coronary syndromes. *Circulation*.

[7] Seizer P., Stellos K., Selhorst G. (2011). CXCL16 is a novel scavenger receptor on platelets and is associated with acute coronary syndrome. *Thrombosis and Haemostasis*.

[8] Yamauchi R., Tanaka M., Kume N. (2004). Upregulation of SR-PSOX/CXCL16 and recruitment of CD8^+^ T cells in cardiac valves during inflammatory valvular heart disease. *Arteriosclerosis, Thrombosis, and Vascular Biology*.

[9] Zivković M., Djurić T., Stojković L. (2015). CXCL16 haplotypes in patients with human carotid atherosclerosis: preliminary results. *Journal of Atherosclerosis and Thrombosis*.

[10] Zhou F., Wang J., Wang K. (2016). Serum CXCL16 as a novel biomarker of coronary artery disease in type 2 diabetes mellitus: a pilot study. *Annals of Clinical and Laboratory Science*.

[11] Laugsand L. E., Åsvold B. O., Vatten L. J. (2016). Soluble CXCL16 and risk of myocardial infarction: The HUNT Study in Norway. *Atherosclerosis*.

[12] Wuttge D. M., Zhou X., Sheikine Y. (2004). CXCL16/SR-PSOX is an interferon-*γ*-regulated chemokine and scavenger receptor expressed in atherosclerotic lesions. *Arteriosclerosis, Thrombosis, and Vascular Biology*.

[13] Lehrke M., Millington S. C., Lefterova M. (2007). CXCL16 is a marker of inflammation, atherosclerosis, and acute coronary syndromes in humans. *Journal of the American College of Cardiology*.

[14] Barrett J. C., Fry B., Maller J., Daly M. J. (2005). Haploview: analysis and visualization of LD and haplotype maps. *Bioinformatics*.

[15] Shi Y. Y., He L. (2005). SHEsis, a powerful software platform for analyses of linkage disequilibrium, haplotype construction, and genetic association at polymorphism loci. *Cell Research*.

[16] Cheng J., Cho M., Cen J.-M. (2015). A TagSNP in SIRT1 gene confers susceptibility to myocardial infarction in a Chinese Han population. *PLoS ONE*.

[17] Seiderer J., Dambacher J., Leistner D. (2008). Genotype-phenotype analysis of the CXCL16 p.Ala181Val polymorphism in inflammatory bowel disease. *Clinical Immunology*.

[18] Huang M., Han Y., Zhang X. (2010). An intron polymorphism in the CXCL16 gene is associated with increased risk of coronary artery disease in Chinese Han population: A lArge Angiography-based Study. *Atherosclerosis*.

[19] Tian J., Hu S., Wang F., Yang X., Li Y., Huang C. (2015). PPARG, AGTR1, CXCL16 and LGALS2 polymorphisms are correlated with the risk for coronary heart disease. *International Journal of Clinical and Experimental Pathology*.

[20] Chua K. H., Ng J. G., Ng C. C., Hilmi I., Goh K. L., Kee B. P. (2016). Association of NOD1, CXCL16, STAT6 and TLR4 gene polymorphisms with Malaysian patients with Crohn's disease. *PeerJ*.

[21] Tatsuguchi M., Furutani M., Hinagata J.-I. (2003). Oxidized LDL receptor gene (OLR1) is associated with the risk of myocardial infarction. *Biochemical and Biophysical Research Communications*.

[22] Zhang Q., Du Y., Zhang J. (2015). Functional impact of 14 single nucleotide polymorphisms causing missense mutations of human *α*7 nicotinic receptor. *PLoS ONE*.

[23] Katoh M. (2008). Cancer genomics and genetics of FGFR2 (Review). *International Journal of Oncology*.

[24] Rebehmed J., Quintus F., Mornon J.-P., Callebaut I. (2016). The respective roles of polar/nonpolar binary patterns and amino acid composition in protein regular secondary structures explored exhaustively using hydrophobic cluster analysis. *Proteins: Structure, Function and Bioinformatics*.

