# Supplementary material for: The Impact of tagSNPs in CXCL16 Gene on the Risk of Myocardial Infarction in a Chinese Han Population
Source: Dis Markers. 2017 Feb 14;2017:9463272. doi: 10.1155/2017/9463272 (PMC5329692; doi:10.1155/2017/9463272)
Supplement: Supplementary file 1 — Table S1 showed the sequences of all the primers and probes used to genotype the four tagSNPs. [file 9463272.f1.doc]

**Table S1 The sequences of the primers and probes used to genotype the SNPs**

| **Name** | **Sequence (5’-3’)** |
| --- | --- |
| **Primers** |  |
| rs2304973-F | GGGGCTGTGGATTGGATG |
| rs2304973-R | CGGGGAGTCGGAAGAAAT |
| rs1050998-F | GCCCACCAGAAGCATTTACT |
| rs1050998-R | TGGATATCTGAAGATGCCCC |
| rs3744700-F | AACCGGCAGATCTGGAAAG |
| rs3744700-R | CACAAATTTTCACTGAGCACCT |
| rs8123-F | CGGGCTTCAGTTTTTCCAT |
| rs8123-R | AGATGGACCCAGAGCTGAGA |
| **Probes** |  |
| rs2304973-FAM | P-GGACGGAGGTGGTCGGCACCTTTTTTTTTTTTTTTTTTTTTTTTTTTTTTTT-FAM |
| rs2304973-C | TTTTTTTTTTTTTTTTTTTTTTTTTTTTTTCCCTCGTTGCCATTGCCTGCGCG |
| rs2304973-T | TTTTTTTTTTTTTTTTTTTTTTTTTTTTTTTTCCCTCGTTGCCATTGCCTGCGCA |
| rs1050998-FAM | P-TTGGGGGGCTGGTAGGAAGTTTTTTTTTTTTTTTTTTTTTTTTTTTTTTTTTTT-FAM |
| rs1050998-C | TTTTTTTTTTTTTTTTTTTTTTTTTTTTTTTTTGCCCCCTCTGAGGCCTGAGAAG |
| rs1050998-T | TTTTTTTTTTTTTTTTTTTTTTTTTTTTTTTTTTTGCCCCCTCTGAGGCCTGAGAAA |
| rs3744700-FAM | P-TCATCCCCCAAACACTGTCCTTTTTTTTTTTTTTTTTTTT-FAM |
| rs3744700-G | TTTTTTTTTTTTTTTTTTATCTGATTCCCTAGTTCAAGCTC |
| rs3744700-T | TTTTTTTTTTTTTTTTTTTTATCTGATTCCCTAGTTCAAGCTA |
| rs8123-FAM | P-ATGAGCACCAACATACCCTGTTTTTTTTTTTTTTTTTTTTTTTTTTTTTTTTTT-FAM |
| rs8123-A | TTTTTTTTTTTTTTTTTTTTTTTTTTTTTTTTTGGGAACATGTAGGGTGGGGAGT |
| rs8123-C | TTTTTTTTTTTTTTTTTTTTTTTTTTTTTTTTTTTGGGAACATGTAGGGTGGGGAGG |
